# Homogenizing solvation: a polarity-gradient strategy for extreme cryogenic batteries

**DOI:** 10.1093/nsr/nwag060

**Published:** 2026-01-31

**Authors:** Zhenyu Guo, Yuanzhu Zhao, Maria-Magdalena Titirici

**Affiliations:** Department of Chemical Engineering, Imperial College London, UK; Department of Chemical Engineering, Imperial College London, UK; Department of Chemical Engineering, Imperial College London, UK; Advanced Institute for Materials Research (WPI-AIMR), Tohoku University, Japan

**Figure 1.**
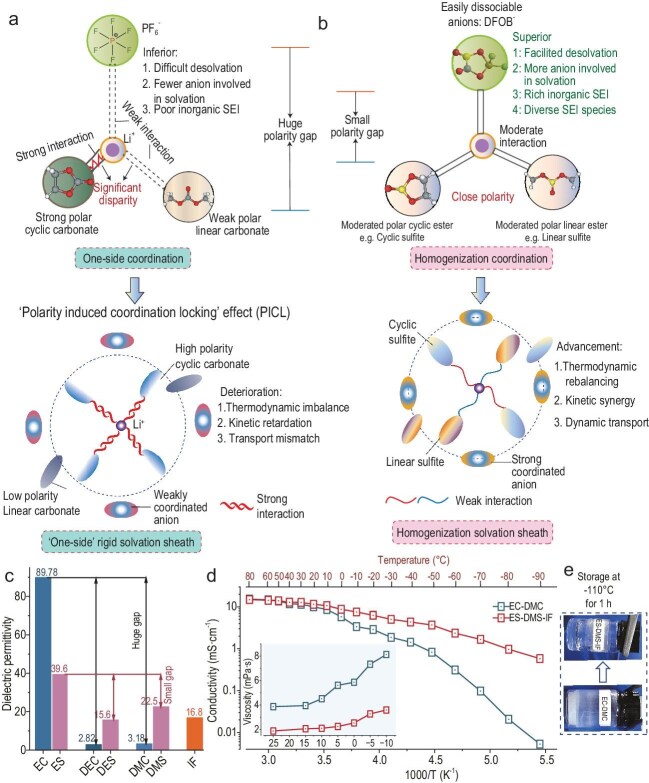
(a) Conventional carbonate electrolytes exhibit high dielectric heterogeneity (∆ε), leading to asymmetric Li⁺ solvation and high desolvation barriers. (b) The PGE strategy minimizes ∆ε through sulfur substitution, enabling balanced coordination among solvents and anions, and facilitating rapid ion transport and stable interphase formation at cryogenic temperatures. (c) Comparison of dielectric permittivity between sulfite and carbonate solvents. (d) Temperature-dependent ionic conductivity. (e) −110°C storage behavior of the electrolytes.

The severe capacity loss of lithium-ion batteries at temperatures below −30°C remains a major obstacle for their use in polar, aerospace and deep-sea scenarios. This persistent failure stems largely from a catastrophic drop in ionic conductivity and sluggish desolvation kinetics [1,2]. In a recent study published in *National Science Review*, Chen *et al*. identify the ‘polarity-induced coordination locking’ (PICL) as the primary failure mechanism in traditional ethylene carbonate (EC)/dimethyl carbonate (DMC) systems [3]. In such mixtures, the imbalanced dielectric contrast in these mixtures creates a segregated environment where high-polarity solvents dominate the inner solvation sheath, leading to a rigid barrier to desolvation. While various strategies such as weakly solvating electrolytes [4] or localized high-concentration designs [5] have attempted to address the cryogenic issue, a critical thermodynamic bottleneck, solvation structure heterogeneity, has largely overlooked, which distorts Li⁺ coordination and interfacial dynamics (Fig. 1a). In contrast to prior approaches that operate within the confines of inherent solvent heterogeneity, the ‘polarity-gradient engineering’ (PGE) strategy represents a paradigm shift by aiming to eliminate this thermodynamic disparity at its source through atomic-scale electronic structure modulation.

To address the PICL issue, Chen *et al.* introduce a PGE paradigm that systematically compresses dielectric disparity via atomic-scale sulfur substitution in carbonate frameworks (Fig. 1b). By replacing carbon with sulfur in cyclic/linear esters, the team reduces ∆ε from 86.6 (EC/DMC) to 17.1 [ethylene sulfite (ES)/dimethyl sulfite (DMS)] (Fig. 1c), thereby balancing Li⁺ coordination among solvents and anions. This homogenized solvation structure enables three synergistic advances: (i) a 45%–56% reduction in desolvation energy barriers (34.97 vs. 79.1 kJ mol^−1^ in carbonates); (ii) formation of an inorganic-rich interphase (LiF/B–O/Li_x_S > 84%) promoted; and (iii) exceptional ionic conductivity (1 mS cm^−1^ at −80°C) (Fig. 1d) with liquid stability down to −110°C (Fig. 1e). This balanced coordination environment actively recruits anions into the primary solvation shell, lowering their reduction potential and thereby steering interfacial decomposition predominantly toward the formation of an inorganic-rich (LiF/B–O/Li_x_S) solid electrolyte interphase (SEI).

The new recipe of a specific electrolyte [1 M lithium difluoro(oxalato)borate (LiDFOB) in ES/DMS/isobutyl formate (IF) (2:4.5:3.5, v/v)] demonstrates remarkable cryogenic performance. In practical 7.5 Ah LiCoO_2_/Li pouch cells, this polyethylene glycol (PEG) electrolyte achieves 81% capacity retention over 400 cycles at −20°C and delivers 73.3% of room-temperature capacity at −60°C. Furthermore, the system demonstrates superior safety, with significantly delayed thermal runaway under abused conditions. While a significant milestone, its commercial viability will need critical techno-economic analysis. Key future work includes developing scalable synthesis routes for battery-grade organic sulfites and LiDFOB, aiming to narrow the cost gap with mature LiPF_6_/carbonate supply chains. Additionally, adapting this strategy to graphite anodes without causing structural degradation will remain a critical goal for the battery community.

In summary, Chen *et al*. provide a molecular blueprint for decoupling the trade-off between ion mobility and desolvation kinetics in cryogenic batteries. Their work establishes a universal ‘polarity gradient—solvation homogeneity—interfacial kinetics’ framework, offering transformative insights for extreme-condition energy storage.
